# Effect of homeopathy on chronic tension-type headache: a pragmatic, randomised controlled single blind trial

**DOI:** 10.1186/1129-2377-14-S1-P56

**Published:** 2013-02-21

**Authors:** N Sharma, A Ameta, S Sharma

**Affiliations:** 1 NMP Medical Research Institute, India; 2 Medical Directorate, Govt. of Rajasthan, India

## Introduction

Homeopathy is increasingly used by headache patients in general practice but scientific evidence is lacking. We therefore designed a clinical trial in a way that would not change the practice pattern of homeopathic physicians.

## Purpose/ background/ objectives

The purpose of the study was to explore individualised homeopathic treatment used in general practice for chronic tension type headache (CCTH).

## Methods

The study was multicentre, pragmatic, randomised controlled trail with blinded assessment. One hundred twenty seven participants with CTTH were randomly assigned to homeopathy or to usual care. Number of headache attacks, duration of pain, pain intensity on visual analog scale, use of medication and resources were recorded through headache diary at 4 weeks run-in-period (baseline), at week 17 post interventions, and end of follow up at week 29. An observer blind to the patients' treatment allocation carried out assessments.

## Results

headache frequency and intensity was lower in the homeopathy group than in controls after intervention (p<0.05) and at follow up (p<0.001). The pain duration was shortened slightly after the intervention period reached to significance level at follow up. In homeopathy group headache parameters decreased at post intervention compared with baseline and continued to decrease slightly in follow up period. The overall evaluation of the 2 treatments indicated improvements in both the treatment but later only homeopathy group showed consistent change. Compared with usual care, patients randomised to homeopathy used 35% less medication (P = 0.001) and had 45% fewer visits to general practitioners (P = 0.0001).

## Conclusion

The results indicate that homeopathy could have clinically relevant benefits for patients with chronic tension type headache.
